# Advancing observer-reported outcome measurement: development of the MOOD-AS for observing distress in Angelman syndrome

**DOI:** 10.1186/s41687-025-00975-1

**Published:** 2025-12-12

**Authors:** Sean N. Halpin, Doesjka A. Hagenaar, Sarah Nelson Potter, Emma van Wooning, Angela Gwaltney, Sabine E. Mous, Anne C. Wheeler

**Affiliations:** 1 GenOmics and Translational Research Center, RTI International, 3040 East Cornwallis Road, Research Triangle Park, Durham, NC 27709, 2194 USA; 2 Department of Child- and Adolescent Psychiatry/Psychology, Erasmus MC, Rotterdam, The Netherlands; 3 Erasmus MC Center of Expertise for Neurodevelopmental Disorders (ENCORE), Erasmus MC, Rotterdam, Netherlands; 4 Carolina Institute for Developmental Disabilities, UNC Chapel Hill, Carrboro, USA

**Keywords:** Outcome measure development, Qualitative methods, Rare disease research

## Abstract

**Background:**

Individuals with Angelman syndrome (AS) often experience profound communication impairment and intellectual disability, and as a result, are often unable to self-report on their experiences of emotional distress, including experiences of anxiety. However, existing measures of anxiety primarily depend on verbal descriptions of internal states, making them unsuitable tools for assessing the nature and severity of distress through observer-report for this population. To address this gap, we developed the Measure of Observable Outcomes of Distress in Angelman Syndrome (MOOD-AS), a novel observer-reported outcome (ObsRO) instrument built through a rigorous, caregiver-centered qualitative process focused on observable expressions of distress.

**Methodology:**

Following FDA and ISPOR guidance for clinical outcome assessment development, we conducted concept elicitation interviews with caregivers in the United States and the Netherlands, drafted an initial item pool, mapped items against existing anxiety measures, and performed cognitive interviews to refine the instrument. Concept elicitation interviews were analyzed using content analysis, with saturation of concept assessed quantitatively. Cognitive interviews focused on evaluating item clarity, relevance, and usability.

**Results:**

Caregivers identified 120 and 119 unique observable behaviors and situations in the U.S. (*n* = 17) and Netherlands (*n* = 8) sample. Saturation of concept was achieved. Mapping to nine existing anxiety measures revealed substantial gaps in coverage of caregiver-observable behaviors. Cognitive interviews (*N* = 9) confirmed strong item clarity and relevance, resulting in minor refinements to instructions, severity ratings, and caregiver impact response options. The final MOOD-AS consists of 71 caregiver-centered items assessing observable distress and anxiety-related behaviors. Psychometric properties will be assessed in the next phase of data collection.

**Conclusions:**

The MOOD-AS provides a rigorously developed ObsRO item set with strong qualitative evidence for content validity. Further psychometric evaluation will establish its measurement properties. Its development highlights the importance of systematically integrating caregiver observations when self-report is not feasible and offers a model for future outcome measure development in rare and nonverbal populations.

**Supplementary Information:**

The online version contains supplementary material available at 10.1186/s41687-025-00975-1.

## Background

Angelman syndrome (AS) is a rare (1:22,000 individuals) [1]; neurogenetic disorder caused by loss of function of the maternal UBE3A gene. The condition is present from birth and typically identified in early childhood due to developmental delays, minimal or absent speech, and motor impairments. Symptoms are lifelong, with functional abilities that remain relatively stable into adulthood rather than showing progressive decline. AS is characterized by severe intellectual disability, limited or absent speech, motor difficulties, seizures, and a complex behavioral phenotype that often includes hyperactivity, sensory processing abnormalities, and traits associated with autism spectrum disorder [2–4]. “Anxiety” has been described as a “frequent” clinical feature in AS through multiple rounds of consensus reporting [3] and almost all caregivers report some behaviors interpreted as anxiety-related distress [5, 6], although these concerns tend to be reported more frequently in persons with AS with the less common non-deletion subtypes of AS (Uniparental disomy, imprinting defects, UBE3A point mutations) [7].

However, assessing the severity and nature of anxiety in individuals with AS presents substantial methodological challenges due to profound communication limitations and cognitive barriers preventing reliable self-report [6]. Standardized anxiety measures, (e.g. the Beck Anxiety Inventory [8]; the Generalized Anxiety Disorder-7 [9]), rely on individuals’ ability to reflect upon and verbalize internal emotional states (e.g., “I feel anxious”), making them unsuitable for individuals unable to articulate such experiences [10]. Observer- or clinician-rated instruments, such as the Aberrant Behavior Checklist (ABC [11]) and the Anxiety, Depression, and Mood Scale (ADAMS [12]), which were designed for individuals with mild to moderate intellectual disability, have shown some utility in assessing emotional-behavioral concerns. Recent work has adapted the ADAMS for broader applicability to individuals with more severe impairment [12], but challenges remain in capturing the nuanced, context-specific behaviors caregivers of individuals with more severe impairments such as AS report [13]. With clinical trials to test disease-modifying therapeutics underway, there is increased emphasis on identifying valid, reliable, and sensitive measures that capture behavioral indicators of distress in this population.

Existing measures are often less suitable for this population because they (1) prioritize internal symptomology inaccessible to third-party observers, (2) inadequately define observable anxiety behaviors, and (3) overlook context-triggered patterns uniquely relevant to neurodevelopmental conditions like AS [14]. Observable behaviors refer to outward, externally verifiable actions visible to an observer, such as trembling, crying, or flinching, rather than inferred emotional states like sadness or fear. Precision in focusing on observable signs is essential for reliability measuring emotional constructs in this population.

Prior research in neurocognitive disorders underscores the methodological importance of emphasizing externally verifiable behaviors, particularly when individuals are unable to self-report, as these behaviors can be reliably observed and rated across caregivers or clinicians. This emphasis also aligns with FDA guidance for observer-reported outcomes, which requires that rated behaviors be visible and objectively verifiable by someone other than the patient [15]. Socio-Emotional Adaptation Theory illustrates how emotional adaptation can be assessed through observable actions in individuals experiencing cognitive decline [16–18]. Similarly, caregiver reports offer crucial insights into emotional states of individuals with significant speech and language difficulties [19, 20].

Robust content validity, the extent to which a measure captures all relevant aspects of a construct for the intended population requires systematic integration of caregiver perspectives [10]. Emotional differences across genetic subtypes, developmental stages, and functional profiles in AS further underscore the need for measures sensitive to individual variation [21]. When rigorously developed, observer-reported outcome (ObsRO) measures provide meaningful tools for capturing distress and anxiety in nonverbal individuals [13, 22].

This manuscript describes the formative process undertaken to develop the *Measure of Observable Outcomes of Distress in Angelman Syndrome (MOOD-AS)*, a caregiver-reported instrument specifically designed to assess observable anxiety-related behaviors. Following U.S. Food and Drug Administration (FDA) and International Society for Pharmacoeconomics and Outcomes Research (ISPOR) guidance for clinical outcome assessment development [10, 15, 23], we applied a three-phase caregiver-centered methodology: (1) concept elicitation interviews to identify observable behaviors, (2) drafting and systematic mapping of items to existing anxiety measures, and (3) cognitive interviews to refine clarity, relevance, and usability. By centering measurement exclusively on caregiver-observed behaviors, the MOOD-AS advances emotional assessment methods for nonverbal and cognitively impaired groups.

## Methods

We followed FDA guidance for developing clinical outcome assessments, focused on observer-reported outcomes (ObsROs), and ISPOR good practices for content validity [10, 15]. These emphasized a patient-centered, qualitative approach to define the concept of interest, identify meaningful outcomes, ensure item relevance, and establish content validity. This study was conducted by rare disease experts [anonymized], USA, in collaboration with the [anonymized], the Netherlands. This international partnership facilitated the inclusion of a large, cross-cultural sample and helped inform a globally relevant measure.

The MOOD-AS was translated from English to Dutch by a professional translation service (Acolad), in consultation with researchers. Translations were reviewed by bilingual Dutch team members to ensure conceptual equivalence and cultural appropriateness; a full forward–back translation was not required at this formative stage. Other study materials used in the Netherlands, including consent forms, demographic questions, interview guides, and data collection templates, were translated by bilingual team members at [anonymized].

### Step 1: concept elicitation interviews

In the concept elicitation interviews, we explored the behaviors caregivers observed in their loved ones with AS, which they attributed to anxiety.

#### Participants and recruitment

U.S. participants were recruited via the Linking Angelman and Dup15q Data for Expanded Research (LADDER) registry [24], and Netherlands participants through the ENCORE expertise center caregiver database and AS-focused community events. All participants completed an online screener to confirm eligibility: (1) parent or legal guardian of someone with AS, (2) perceived anxiety-related behaviors, (3) age 18+, (4) fluency in English (U.S.) or Dutch (NL), (5) internet and webcam access, and (6) willingness to be audio recorded. The screener included an open-ended item (“Please describe one way your loved one expresses anxiety”) used to personalize interviews, and a family history question on anxiety disorders.

Eligible participants provided verbal consent and completed a demographic form. To ensure diversity, we recruited at least 50% of participants with loved ones diagnosed with deletion subtype AS, given its distinct behavioral profile [7], and purposively sampled for age variation to capture anxiety behaviors across developmental stages. Interviews were scheduled via Microsoft Bookings (U.S.) or phone (NL). Each participant completed both a concept elicitation and cognitive interview.

#### Interview procedures

Interviews were conducted virtually via Microsoft Teams (U.S.: SNH, SA; NL: DH), except for one in-person interview in the Netherlands due to participant preference. A semi-structured guide was used to elicit caregiver-identified behaviors associated with perceived anxiety, structured in two parts. The first part focused on spontaneous reporting, referencing screener responses and inviting caregivers to describe additional behaviors, recognition cues, evolution over time, and impacts on daily life—allowing for participant-led narratives consistent with concept elicitation guidance [25].

The second part used structured probes from the Behaviors Indicating Anxiety in People with Angelman Syndrome (BIAPAS), which includes 27 situations and 67 behaviors, encompassing all 28 ADAMS items. Caregivers were asked whether each item applied to their loved one, whether it reflected anxiety, and why. Probes included both situational triggers (e.g., “during transitions,” “at a doctor’s office”) and behavioral signs (e.g., “avoids eye contact,” “trembles when frightening situations are not present”). All interviews were audio-recorded and transcribed via Microsoft Teams. Each transcript was reviewed by a member of the qualitative research team against the original audio recording to confirm accuracy and correct transcription errors before coding and analysis. Dutch interviews were translated into English for analysis and reviewed for accuracy.

#### Data analysis

We analyzed interview data using Krippendorf’s content analysis framework, which emphasizes systematic interpretation of meaning from text via defined units of analysis [26]. Each distinct anxiety-related behavior described by caregivers was treated as a recording unit, with each participant as a separate context unit in a matrix. The matrix was initially populated from structured interview notes and supplemented with transcript quotes to ensure fidelity to participant language.

SNH, SA, and SNP independently reviewed participant quotes to inductively identify externally verifiable behaviors (e.g., “tearful” instead of inferred states like “sad”). Behaviors were iteratively grouped into emergent themes.

We separated behaviors into distinct items when they reflected clearly different observable manifestations (e.g., freezing vs. crying) or conveyed different emotional or physical responses. Minor language variations (e.g., “stiff” vs. “frozen”) were combined. Final decisions about item inclusion, splitting, or merging were made via team consensus meetings, with review of original quotes to preserve caregiver intent. Ambiguities were adjudicated based on context and caregiver phrasing [27].

Saturation was assessed using the ratio of new behaviors identified in the final interview to the cumulative total across all interviews [28]. A near-zero saturation ratio indicated that core behavioral concepts were comprehensively captured and that further interviews were unlikely to yield substantial additions.

### Step 2: drafting an initial item pool and mapping draft to existing measures

Following concept elicitation analysis, we developed a draft pool of MOOD-AS items representing distinct, observable behaviors that caregivers associated with anxiety in individuals with AS. Observable behaviors were defined as those that could be seen or heard by an outside observer (e.g., “whining,” “pacing,” “crying”), in contrast to inferred emotional states (e.g., “feels anxious,” “is worried”).

To evaluate the novelty and comprehensiveness of this draft pool, we mapped each item against existing anxiety-related instruments to identify conceptual overlap and, critically, gaps- particularly with respect to observable indicators in nonverbal individuals.

Table 1 presents the instruments reviewed. Most traditional anxiety tools rely on patient-reported internal states, limiting their relevance for nonverbal populations. In contrast, a few informant- or clinician-rated tools (e.g., ABC, ADAMS) include observable items but do not provide full coverage of behaviors relevant to AS. MOOD-AS was intentionally constructed to fill this gap by focusing exclusively on caregiver-observed signs of distress in individuals with limited expressive communication.

This mapping exercise confirmed that MOOD-AS addressed a critical measurement need and ensured its items were both distinct from and complementary to existing scales, grounded in the lived experiences of families.

**Table 1:** Anxiety-related instruments reviewed in mapping exercise

Instrument	Reporter Type	Includes Observable Items	Notes
Aberrant Behavior Checklist (ABC) [11]	Caregiver	Yes	Widely used in neurodevelopmental contexts
Anxiety, Depression, and Mood Scale (ADAMS) [12]	Caregiver	Yes	Includes multiple observable items
Behaviors Indicating Anxiety in People with Angelman Syndrome (BIAPAS) (unpublished) (supplementary file 1)	Caregiver/Clinician	Yes	Used in this study for structured probing
Hamilton Anxiety Rating Scale (HAM-A) [29]	Clinician	Partially	Includes some externally rated signs
Neuropsychiatric Inventory Questionnaire (NPI-Q) (Kaufer et al., 2000)	Caregiver	Partially	Targets general neuropsychiatric symptoms
Penn State Worry Questionnaire (PSWQ) [30]	Patient	No	Focused on internal cognitive states
Beck Anxiety Inventory (BAI) [8]	Patient	Partially	Includes some somatic signs like trembling
Generalized Anxiety Disorder-7 (GAD-7) [9]	Patient	No	Internal worry and emotional states only
State-Trait Anxiety Inventory (STAI) [31]	Patient	No	Assesses self-perceived anxiety

### Step 3: cognitive interviews to refine the instrument

The draft version of the MOOD-AS, consisting of 71 items derived from caregiver input and refined through concept mapping, was then subjected to cognitive testing to evaluate its clarity, relevance, and usability. Although presented here as discrete steps, development of the MOOD-AS was iterative, and feedback from cognitive interviews also informed minor refinements to earlier content.

#### Participants and recruitment

We followed the same recruitment and eligibility procedures as in Step 1. Cognitive interviews were conducted with nine caregivers (four in the United States and five in the Netherlands), consistent with established methodological guidelines for cognitive interview studies in clinical outcome assessment [23, 32]. The purpose of cognitive interviewing is not thematic saturation, but to assess respondents’ understanding of items, instructions, and response scales, and to identify confusion, ambiguity, or misinterpretation [32, 33]. Standards recommend small, purposive samples, typically five to fifteen participants—as sufficient to detect major issues, with limited added benefit beyond that range [33]. Our nine participants fall well within this recommended range, balancing efficiency with diversity.

U.S. participants were recruited via the LADDER database [24]; Netherlands participants were recruited from an existing caregiver database and AS-focused community events. All completed the same screener, consent, and demographic form used in Step 1, and were invited to a second interview focused on refining the MOOD-AS. As before, we purposively recruited caregivers to ensure that at least 50% reported a deletion subtype and that participant age varied to reflect the developmental range of individuals with AS.

#### Interview procedures

Cognitive interviews were conducted virtually using Microsoft Teams (U.S.: SNH, SA; NL: DH, EvW), and with one conducted in person, following a structured guide focused on reviewing the draft MOOD-AS. Participants completed the 71 item, draft measure in advance or during the session. Interviews were audio-recorded, transcribed verbatim, and participants were encouraged to “think aloud” as they reviewed items to facilitate exploration of their reasoning and interpretation. Caregivers were instructed to rate each observable behavior based on how often and how severe it had been during the past four weeks. Interviewers then guided them through structured feedback on the domains in Table 2. This structured format allowed caregivers to reflect not only on individual items but on the overall utility and comprehensibility of the measure in relation to their lived experience.

**Table 2:** Cognitive interview domains assessed in review of the draft MOOD-AS

Domain	Description
Title	Caregivers were asked which of three titles best reflected the measure’s purpose: Measure of Observable Outcomes of Distress in Angelman Syndrome (MOOD-AS), Measure of Expressive Behaviors in Angelman Syndrome (MEB-AS), and Angelman Syndrome Behavioral Signals Index (AS-BSI).
Instructions and Response Scales	Participants reviewed and discussed the clarity of the measure’s instructions and the two rating scales, frequency (never, rarely, sometimes, often) and severity (mild, moderate, severe, very severe).
Items	Each of the 71 items was reviewed for clarity and relevance using a 4, point scale (not at all clear or relevant to very clear and relevant), followed by open, ended suggestions. Targeted probes were used for potentially redundant or unclear items (e.g., “reduced communication” vs. “decreased communication”).
Final Items	The last two items on behavioral context and caregiver impact were reviewed for clarity and completeness.
Overall Impressions	Interviews ended with an open prompt for final thoughts on the measure as a whole.

#### Data analysis

Interviewers used a structured notes template to document feedback in real time. After interviews concluded, the research team reviewed and coded all feedback related to item clarity, relevance, suggested revisions, and preferred title. Items repeatedly flagged as unclear, redundant, or difficult to observe were revised, consolidated, or removed. Minor wording changes were made to improve readability and translation compatibility. Revisions were made through team consensus and grounded in participant feedback, reflecting best practices in instrument refinement and cognitive testing [25, 32, 34]. Items that multiple participants identified as unclear, redundant, or difficult to observe were prioritized for revision or removal, with decisions balancing participant input and conceptual coverage. This process resulted in a streamlined and caregiver-centered version of the MOOD-AS, ready for future quantitative validation.

## Results

### Participants

Table 3 summarizes demographic and clinical characteristics of participants in the concept elicitation (*N* = 25) and cognitive interviews (*N* = 9). Most caregivers identified as female, with ages ranging from 31 to 80 years. Education levels varied across countries, with higher levels of vocational training reported in the Netherlands. Persons with AS ranged from 3 to 56 years old, with deletion being the most reported genotype, though under 50% in the cognitive sample. A family history of anxiety was reported by over half of concept elicitation participants but only 22% in cognitive interviews. These samples reflected relevant diversity in caregiver and AS characteristics for the intended measure.

**Table 3:** Participant characteristics

Characteristic	Concept elicitation interviews			Cognitive interviews		
	US (*n* = 17)	Netherlands (*n* = 8)	Total (*N* = 25)	US (*n* = 4)	Netherlands (*n* = 5)	Total (*N* = 9)
**Interview length (minutes) Median (Min-Max)**	70 (36–97)	75 (70–99)	74 (36–99)	76 (64–97)	84 (57–105)	83 (57–105)
**Caregiver’s Age (years) Median/Range**	45 (31–68)	53 (32–80)	45 (31–80)	52 (41–67)	42 (32–70)	43 (32–70)
**Caregiver’s Sex**						
Female	15 (88%)	7 (88%)	22 (88%)	3 (7%)	5 (100%)	8 (89%)
Male	2 (12%)	1 (12%)	3 (12%)	1 (25%)	0 (0%)	1 (11%)
**Caregiver’s Race**						
American Indian or Alaska Native	1 (6%)	NR	1 (6%)	0 (0%)	NR	0 (0%)
Asian	1 (6%)	NR	1 (6%)	0 (0%)	NR	0 (0%)
White	15 (88%)	NR	15 (88%)	4 (100%)	NR	4 (100%)
**Caregiver’s Education (US)**						
Some college or technical school	3 (18%)	NR	3 (18%)	0 (0%)	NR	0 (0%)
Graduated: Bachelor’s degree (B.A./B.S.)	8 (47%)	NR	8 (47%)	2 (50%)	NR	2 (50%)
Graduated: Master’s/Other graduate degree	6 (35%)	NR	6 (35%)	2 (50%)	NR	2 (50%)
**Caregiver’s Education (Netherlands)**						
Lower level secondary school	NR	2 (25%)	2 (25%)	NR	1 (20%)	1 (20%)
Lower vocational education	NR	2 (25%)	2 (25%)	NR	1 (20%)	1 (20%)
Higher vocation education	NR	2 (25%)	2 (25%)	NR	2 (40%)	2 (40%)
University bachelor en/of master	NR	1 (13%)	1 (13%)	NR	1 (20%)	1 (20%)
University PhD	NR	1 (13%)	1 (13%)	NR	0 (0%)	0 (0%)
**Anxiety or Anxiety Disorder Family History**						
Yes	12 (71%)	2 (25%)	14 (56%)	2 (50%)	0 (0%)	2 (22%)
No	5 (29%)	4 (50%)	9 (36%)	2 (50%)	4 (80%)	6 (67%)
Not answered	0 (0%)	2 (25%)	2 (8%)	0 (0%)	1 (20%)	1 (11%)
Relationship of person with anxiety to person with AS*						
Parent or guardian	10 (59%)	7 (88%)	17 (68%)	0 (0%)	0 (0%)	0 (0%)
Sibling	4 (24%)	1 (13%)	5 (20%)	1 (25%)	0 (0%)	1 (11%)
Grandparent	2 (12%)	0 (0%)	2 (8%)	0 (0%)	0 (0%)	0 (0%)
Uncle/Aunt	2 (12%)	0 (0%)	2 (8%)	0 (0%)	0 (0%)	0 (0%)
Other	4 (24%)	0 (0%)	4 (16%)	3 (75%)	5 (100%)	8 (89%)
**Person with AS’s Age (years) Median/Range**	15 (3–32)	17 (3–56)	15 (3–56)	8 (6–30)	9 (3–39)	9 (3–39)
**Person with AS’s Sex**						
Female	9 (53%)	5 (63%)	14 (56%)	3 (75%)	4 (80%)	7 (78%)
Male	8 (47%)	3 (38%)	11 (44%)	1 (25%)	1 (20%)	2 (22%)
**Person with AS’s Race**						
American Indian or Alaska Native	1 (6%)	NR	1 (6%)	0 (0%)	NR	0 (0%)
Asian	2 (12%)	NR	2 (12%)	0 (0%)	NR	0 (0%)
Black	2 (12%)	NR	2 (12%)	0 (0%)	NR	0 (0%)
Hispanic	3 (18%)	NR	3 (18%)	1 (25%)	NR	1 (25%)
White	11 (65%)	NR	11 (65%)	3 (75%)	NR	3 (75%)
**Mutation subtype**						
Deletion	9 (53%)	4 (50%)	13 (52%)	1 (25%)	2 (40%)	3 (33%)
Paternal uniparental disomy	2 (12%)	1 (13%)	3 (12%)	1 (25%)	1 (20%)	2 (22%)
Imprinting defect	3 (18%)	1 (13%)	4 (16%)	1 (25%)	1 (20%)	2 (22%)
UBE3A mutation	3 (18%)	2 (25%)	5 (20%)	1 (25%)	1 (20%)	2 (22%)
**Mosaicism status**						
Yes	3 (18%)	0 (0%)	3 (12%)	1 (25%)	0 (0%)	1 (11%)
No	12 (71%)	3 (38%)	15 (60%)	3 (75%)	2 (40%)	5 (56%)
Unsure	2 (12%)	5 (62%)	7 (28%)	0 (0%)	3 (60%)	3 (33%)

Note: Percentages are calculated within each country and interview type. “NR” indicates the item was not reported in that country

*Participants could select more than one family member with a history of anxiety or anxiety disorder

** In Netherlands for one interview each phase, both parents participated in the interview. We report on only the mother’s demographics in the table above for that interview

### Concept elicitation results

In total, 120 unique behaviors and situations were identified across U.S. participants, with a median of 31 behaviors reported per participant (IQR = 6–69). Among Netherlands participants, 119 unique behaviors and situations were identified, with a median of 25 behaviors per participant (IQR = 9–36). Saturation of concept was achieved in both countries, with a ratio of 0.008 in the United States and 0.042 in the Netherlands.

Many caregiver descriptions directly confirmed observable behaviors that required no modification from the original *BIAPAS* items. For example, the item *tense* was endorsed by 11 U.S. caregivers (34%) and 6 Netherlands caregivers (75%), with descriptions such as: *“Frozen*,* your muscles are all like this and stiff”* (U.S. Participant 43). Similarly, the item *bites or tries to bite someone* was endorsed by 5 U.S. caregivers (29%) and 1 Netherlands caregiver (13%), with one explaining: *“He bites and it’s always the same. It’s the same spot on his arm*,* and it’s just like he’ll just kind of lean over and bite his arm”* (U.S. Participant 32).

In other cases, endorsement of a single *BIAPAS* item, such as ‘at the doctor’s office’, revealed multiple distinct observable behaviors, prompting refinement into separate draft items for the MOOD-AS. For instance, this situation was endorsed by 8 U.S. caregivers (47%) and all Netherlands caregivers (100%). Participant descriptions included: *“she freezes*,* her whole body just stiffens*,*” “her eyes widen*,*” “she starts crying*,*”* and *“she flaps her hands”* (U.S. Participant 4). One caregiver added: *“You hold his arms and I hold his legs. He still manages to get away… he flinches*,* trembles*,* and tries to run”* (Netherlands Participant 1). These behaviors were split into distinct MOOD-AS items to support more precise and actionable measurement.

These examples guided decisions about whether a behavior could remain unchanged or should be refined into more specific observable indicators. Even behaviors endorsed by only a few caregivers were retained if they were described with consistent language and clearly distinguished during analysis as relevant expressions attributed to anxiety.

### Drafting an initial item pool and mapping draft to existing measures results

Following concept elicitation interviews, we generated an initial pool of 69 distinct observable behaviors caregivers attributed to anxiety in individuals with AS. Two additional items were added to assess (1) the context in which behaviors occurred and (2) the perceived impact on the caregiver, resulting in a 71-item draft of the MOOD-AS.

To assess novelty and coverage, we mapped each draft item against nine existing anxiety-related measures including observer-, clinician-, and self-reported measures, including the Aberrant Behavior Checklist (ABC), Anxiety, Depression, and Mood Scale (ADAMS), Behavior Indicating Anxiety in People with Autism Spectrum Disorder (BIAPAS), Hamilton Anxiety Rating Scale (HAM-A), Neuropsychiatric Inventory–Questionnaire (NPI-Q), Penn State Worry Questionnaire (PSWQ), Beck Anxiety Inventory (BAI), Generalized Anxiety Disorder 7-item scale (GAD-7), and State-Trait Anxiety Inventory (STAI), to identify overlapping and unique content. Each measure was reviewed for externally observable items—those a third party could see or hear. Items like “wobbliness in legs” (BAI) were included, while internal-only items (e.g., “I tend to worry about things” [PSWQ]) were excluded. The proportion of observable items ranged from 0% to 100% across measures. Widely used self-report tools (e.g., PSWQ, GAD-7) lacked observable items, while caregiver- and clinician-informed tools (e.g., ABC, ADAMS) included more—but still showed gaps. This inconsistency highlighted the need for a caregiver-focused tool grounded in directly observable behaviors. Across all nine instruments reviewed, fewer than one-third of items mapped directly onto observable expressions of distress, underscoring the limited content coverage of observable anxiety behaviors in existing measures.

Most reviewed instruments, especially patient-reported measures (PSWQ, BAI, GAD-7, STAI), emphasized internal states. Observable behaviors were more common in clinician- or observer-rated tools (HAM-A, NPI-Q, ABC, ADAMS, BIAPAS) but still lacked many caregiver-identified signs such as flinching, stiffening, and context-specific aggression (e.g., during medical procedures). We also drew from these instruments to inform MOOD-AS item structure, response scales, and instructions. Frequency-based scales (e.g., ABC) and severity anchors (e.g., HAM-A) influenced the format, while language was refined for plain-language readability and caregiver usability.

In sum, the mapping confirmed the need for a new ObsRO focused solely on observable behaviors associated with anxiety-related distress in AS. The MOOD-AS was developed to address this gap.

### Cognitive interviews to refine the instrument results

The goal of the cognitive interviews was to evaluate the, relevance, and usability of the draft MOOD- AS. Nine caregivers participated, four from the United States and five from the Netherlands, reviewing the measure’s title, instructions, response options, and items. Participant feedback informed refinements to the MOOD-AS. A summary of feedback and resulting revisions is presented in Table 4.

#### Title and instructions

Caregivers in both countries generally endorsed the title *Measure of Observable Outcomes of Distress in Angelman Syndrome (MOOD-AS)* as clearly reflecting the purpose of the instrument. A few Netherlands caregivers found the title long but ultimately understood its meaning and preferred it over the alternatives. One participant noted, *“It makes it clear that it’s not just anxiety but any form of distress*,* emotional or situational”* (US Participant 7). In response to feedback from both countries, the instructions were revised to emphasize earlier that caregivers should report only observable behaviors, not inferred internal states.

#### Frequency and severity questions

The frequency question (“How often did you observe this behavior in the last 4 weeks?”) was considered clear and easy to respond to by all participants. No revisions were made. However, the severity question prompted uncertainty in both countries regarding whether it referred to the person with AS’s distress or the caregiver’s experience. One caregiver explained, *“I wasn’t sure*,* do you mean how bad it was for her*,* or how much it disrupted our day?”* (Netherlands Participant 6). To clarify, the item was revised to specify that caregivers should rate the severity for the person with AS.

#### Behavioral items

Across interviews, the 69 behavioral items were rated as clear and relevant. Several caregivers emphasized that they could easily recall instances of these behaviors in daily life. One US caregiver stated, *“All of these are things I have seen*,* maybe not all in the last month*,* but definitely over time”* (US Participant 7). No changes were made to the behavioral items.

#### Context and impact items

Both sets of context and impact items were reviewed positively. However, participants suggested that the impact scale (for caregiver experience) could benefit from **more distinct wording** between “slight,” “moderate,” and “significant.” One U.S. participant noted: *“Sometimes it’s hard to know the difference between moderate and significant*,* maybe give examples?”* (US Participant 43). We refined the response scale to clarify anchor definitions.

**Table 4:** Summary of cognitive interview feedback and revisions to the MOOD-AS

Section evaluated	Feedback summary	Action taken
Title	Well, received across both countries; minor concern about length	No change
Instructions	Suggest emphasizing “observable behaviors” earlier	Revised text
Frequency Question	Clear and easy to understand	No change
Severity Question	Clarify severity is about loved one with AS, not caregiver impact	Revised question wording
Behavioral Items	Clear and relevant; no changes suggested	No change
Context Items	Clear and relevant	No change
Impact Items	Minor suggestion to clarify scale wording	Revised response scale wording

Based on this feedback, the MOOD-AS was finalized with minor edits to the instructions, severity question, and caregiver impact response options. No behavioral items were added or removed. The instrument is now ready for formal psychometric validation in a larger sample.

### Evolution of items across developmental phases

After completing cognitive interviews and finalizing the MOOD-AS, we compared the original BIAPAS items, concept elicitation findings, and final MOOD-AS wording to illustrate how specific behaviors were refined over the course of measure development. Table 5 provides illustrative examples of item evolution across development stages.

**Table 5:** Example item evolution across development stages

BIAPAS Item/Trigger	Concept elicitation outcome	Initial MOOD-AS item	Final MOOD-AS item
Tense	Confirmed observable (“frozen,” “stiff”)	Increased tensing of their body	Same (no change)
At doctor’s office	Multiple behaviors identified (freezing, running, crying)	Split into separate items: (1) Increased tensing of their body; (2) Flight behavior (elopes, runs away, or wanders away); (3) tearful	Same (no change)
Bites or tries to bite someone	Confirmed behavior with consistent examples	Bites or tries to bite someone	Same (no change)
Trembles when frightening situations are not present	Confirmed across settings (doctor’s office, crowded areas)	Increased trembling even when frightening situations are not present	Same (no change)
Avoids eye contact	Confirmed as an anxiety response	Avoids eye contact	Same (no change)

## Discussion

This study describes the rigorous development of the MOOD-AS, a novel observer-reported outcome (ObsRO) measure designed to capture caregiver-observable indicators of anxiety-related distress in individuals with AS. Consistent with best practice guidance from the FDA [15] and ISPOR [10], we employed a multi-step, qualitative methodology emphasizing concept elicitation, iterative item refinement, and cognitive interviewing. The resulting measure addresses longstanding challenges in assessing emotional states in nonverbal and cognitively impaired populations.

Unlike traditional anxiety instruments that rely on self-reported internal experiences (e.g., “I feel worried”), the MOOD-AS was explicitly designed to assess externally verifiable, observable behaviors. Concept elicitation interviews yielded 120 and 119 unique observable behaviors in U.S. and Netherlands samples, respectively, providing strong initial content richness. Observable indicators such as “freezing,” “crying,” “stiffening,” and “flinching” were consistently reported, reflecting concrete manifestations of distress that align with recent calls for the development of caregiver-centered emotional measures in neurodevelopmental disorders [6, 20].

Mapping against existing anxiety instruments revealed that few measures, whether patient-, clinician-, or observer-reported, captured the observable behaviors identified through caregiver interviews. Most standardized tools, including the Beck Anxiety Inventory [8] and the Generalized Anxiety Disorder-7 [9], emphasize internal cognitive-affective states that cannot be reliably observed by third parties [10, 19]. Even clinician-rated scales like the Hamilton Anxiety Rating Scale [29] and Neuropsychiatric Inventory Questionnaire [35] did not capture many of the behaviors identified through caregiver interviews. These findings reinforce the methodological need, articulated in outcome assessment development literature to create ObsRO measures that define observable behaviors as the unit of analysis when self-report is unattainable [23, 36].

Cognitive interviews with nine caregivers confirmed the face validity, clarity, and relevance of the MOOD-AS items. This small, purposively selected sample aligns with methodological standards for cognitive interviewing in instrument development [23, 32, 33], where the goal is refinement of item interpretation rather than concept discovery. Participant feedback prompted minor but meaningful revisions to the instructions, severity question wording, and caregiver impact response scale, further strengthening the instrument’s usability. The absence of major item removal or restructuring provides additional support for the robustness of the initial item generation process.

Importantly, the MOOD-AS development approach contributes methodologically by operationalizing emotional assessment through observable, caregiver-interpreted behaviors, a model that may be extended beyond AS. Prior conceptual frameworks, including Socio-Emotional Adaptation Theory [16–18], emphasize that when direct self-report is compromised, externally verifiable behavioral expressions offer the most ethically and scientifically sound basis for inferring emotional experiences. Our work advances this model by embedding it into a formal ObsRO measure specifically designed for clinical research use.

Beyond AS, the MOOD-AS process offers a methodological blueprint for developing ObsRO instruments across other neurodevelopmental and cognitively impaired populations, such as individuals with Rett syndrome, Phelan-McDermid syndrome, or advanced Alzheimer’s disease. In these populations, internal emotional states are similarly inaccessible, and observable, caregiver-interpreted behaviors represent the primary data source for understanding emotional health. The integration of caregiver expertise, careful separation of observable behaviors from inferred states, and iterative refinement through cognitive interviewing exemplify best practices that could elevate the content validity of outcome assessments across a wide range of clinical contexts. Future measure development efforts should prioritize observable, contextually grounded behaviors and systematically incorporate caregiver input to achieve greater validity and applicability for populations with profound communication limitations.

Several limitations should be acknowledged. First, although saturation of concept was quantitatively achieved [28], our sample was modest and primarily drawn from research-engaged caregiver populations, which may limit the generalizability of findings to more diverse or less research-involved families. Most participating caregivers identified as White and had relatively high levels of education. Although common in rare-disease research, future work should prioritize inclusion of caregivers from racially, ethnically, and socioeconomically diverse backgrounds to support broader generalizability and acceptability of the MOOD-AS. Second, all data regarding observable behaviors were derived from caregiver report, introducing the potential for subjective bias in how behaviors were interpreted or recalled. This includes possible influence from the caregiver’s own mental health history, such as a family history of anxiety, which may shape how they perceive and attribute distress behaviors in their loved one with AS. Beyond potential influence from caregivers’ own mental health history, factors such as caregiver stress, expectations, and cultural framing may also shape how behaviors are perceived and described. These influences do not diminish the value of caregiver insights but highlight the importance of understanding the contexts in which proxy reports are generated. Future work should examine how these factors may affect interpretation and content validity. Additionally, caregivers may adapt to their child’s characteristic behaviors over time, potentially leading to underrecognition or normalization of behaviors that reflect distress or anxiety. Future research should explore how caregiver adaptation influences interpretation of observable behaviors and whether this affects sensitivity to change. While this factor was self-reported during screening, its potential influence on caregiver interpretation introduces a circular challenge that future research should explore. While caregivers offer critical insights, future research should explore triangulating caregiver-reported behaviors with other data sources, such as clinician observations, ecological momentary assessments, video-based behavioral coding, or biological and physiological measures such as cortisol levels, to strengthen external validation. Third, although cross-national sampling improved the linguistic and cultural robustness of the MOOD-AS, formal measurement invariance testing is needed to assess whether the instrument performs consistently across different cultural and language groups. Because of the limited number of eligible caregivers available in this rare disease population, some participants took part in both the concept elicitation and cognitive interview phases. Although separate samples could have provided broader perspectives, this approach is common and appropriate in rare disease contexts to ensure adequate representation. Finally, although cognitive interviews supported content validity, further psychometric evaluation, including assessments of internal consistency, test-retest reliability, construct validity, and sensitivity to change, is required before the MOOD-AS can be recommended for regulatory or clinical trial use. Psychometric evaluation will apply both Classical Test Theory and Item Response Theory approaches to examine item performance, reliability, and dimensionality. Items showing limited variability, weak correlations, or redundancy will be reviewed for potential removal to produce a streamlined, psychometrically robust measure.

Future research should prioritize psychometric validation of the MOOD-AS and explore its ability to differentiate distress-related behaviors from other causes of behavioral dysregulation, such as pain or gastrointestinal discomfort. This may involve integrating caregiver-report data with clinician observations, behavioral coding, or physiological measures (e.g., cortisol levels) to improve specificity and reduce attribution error. A follow-up study incorporating cortisol sampling is currently planned and may offer additional insight into how observed behaviors align with underlying biological stress responses. Moreover, the MOOD-AS offers a concrete model for ObsRO development in other rare diseases, particularly where meaningful caregiver-centered endpoints are increasingly emphasized [13, 22].

In summary, the MOOD-AS represents an important methodological and clinical advance in the assessment of anxiety-related distress in AS. By grounding measurement exclusively in observable caregiver-reported behaviors and applying rigorous ObsRO development standards, this study contributes a replicable, ethically grounded, and scientifically rigorous model for emotional assessment in populations where traditional psychiatric approaches fail.

## Supplementary Information

Below is the link to the electronic supplementary material.

Supplementary Material 1

## Data Availability

Data from this study are not available for sharing.

## Abbreviations

ABC: Aberrant Behavior Checklist
ADAMS: Anxiety, Depression, and Mood Scale
AS: Angelman syndrome
AS-BSI: Angelman Syndrome Behavioral Signals Index
BAI: Beck Anxiety Inventory
BIAPAS: Behaviors Indicating Anxiety in People with Angelman Syndrome
FDA: U.S. Food and Drug Administration
GAD-7: Generalized Anxiety Disorder 7-item scale
HAM-A: Hamilton Anxiety Rating Scale
ISPOR: International Society for Pharmacoeconomics and Outcomes Research
LADDER: Linking Angelman and Dup15q Data for Expanded Research
MOOD-AS: Measure of Observable Outcomes of Distress in Angelman Syndrome
NPI-Q: Neuropsychiatric Inventory Questionnaire
ObsRO: Observer-Reported Outcome
PSWQ: Penn State Worry Questionnaire
STAI: State-Trait Anxiety Inventory
U.S.: United States
