# Supplementary material for: Advancing observer-reported outcome measurement: development of the MOOD-AS for observing distress in Angelman syndrome
Source: J Patient Rep Outcomes. 2025 Dec 12;10:8. doi: 10.1186/s41687-025-00975-1 (PMC12816459; doi:10.1186/s41687-025-00975-1)
Supplement: Supplementary file 1 — Supplementary Material 1 [file 41687_2025_975_MOESM1_ESM.pdf]

Behaviors Indicating Anxiety in People with Angelman Syndrome *(BIAPAS)*

Modified from the Carolina Institute for Developmental Disabilities
Angelman Syndrome Questionnaire and the ADAMS

Created by Kunz P.A., Jones K.A., Berry S., Christian R., Wheeler A.
Modified by Wheeler A, Bichell TJ, Bird L, Sadwhani A,, Keary C and Tonnsen B

Child's Name____

Birthdate______________ Today's Date____________

Sex____________ Race/Ethnicity_________________

Name of Person(s) Completing Questionnaire_________________________

Relationship to person with AS_____________________________________

4 - What is the genotype of the person with Angelman syndrome? Circle one:

Deletion UBE3A mutation Imprinting Defect Uniparental Disomy

Other, please specify____

5 - If the person has a deletion, what is the size of the deletion? Circle one:

Class I Class II Class III Class IV Class V Unknown

6. In general, do you think the person with Angelman syndrome is anxious? ___Yes ___No

If yes, circle one: occasionally sometimes frequent almost always

If yes, how concerning is the anxiety to you? Please describe:_______________

____

____

PART 1. CAREGIVER IMPRESSIONS OF ANXIETY:

1. In general, how often does the person with Angelman syndrome display anxious behaviors?

Never Rarely Sometimes Often Always

2. How much does anxiety interfere with the quality of life of the person with Angelman syndrome?

Never Rarely Sometimes Often Always

3. How often does the family adapt life to minimize anxiety symptoms?

Never Rarely Sometimes Often Always

PART 2: A PERSON'S SPECIFIC ANXIETY BEHAVIORS

1. In general, how can you tell when the person with AS is anxious?

2. Please rank the top 5 situations in which you believe the person with AS displays anxiety (e.g. during transitions, around animals) and how they show anxiety in that situation (e.g. crying, increased laughter).

	Situation	Behaviors
1		
2		
3		
4		
5		

3. Please look through the list below and indicate the situations in which a behavior has occurred.

	Situation	Yes	No	Not applicable
1	During transitions			
2	Entering a restaurant			

3	Waiting for food at a restaurant			
4	Waiting for food at home			
5	Getting off a school bus to school			
6	Getting off a school bus at home			
7	Welcoming guests			
8	With new people			
9	In a new activity			
10	When a parent departs			
11	When a caregiver departs			
12	When a preferred person departs			
13	At a doctor’s office			
14	Getting a shot or blood test			
15	Getting nails clipped			
16	In a noisy place			
17	In the car			
18	In a crowd			
19	In a confined space			
20	When a preferred person moves in a room			

21	When a preferred object is removed			
22	Around animals			
23	At bedtime			
24	On a roller coaster			
25	In the bathroom			
26	Always			
27	Unable to predict situation			
28	Other, please specify			
29	Other, please specify			
30	Other, please specify			

4. Please circle the response that best describes the severity of the person with AS's anxiety behavior in the situations you indicated.

(0) not a problem (1) mild problem (2) moderate problem (3) severe problem

						In which situations?
1	Nervous	0	1	2	3	
2	Problem initiating communication	0	1	2	3	
3	Does not relax or settle down	0	1	2	3	
4	Has periods of over-activity	0	1	2	3	

5	Sleeps more than normal	0	1	2	3	
6	Withdraws from other people	0	1	2	3	
7	Tense	0	1	2	3	
8	Engages in ritualistic behaviors	0	1	2	3	
9	Depressed mood	0	1	2	3	
10	Sad	0	1	2	3	
11	Worried	0	1	2	3	
12	Has developed difficulty staying on task or completing work	0	1	2	3	
13	Shy	0	1	2	3	
14	Easily fatigued (not due to being overweight)	0	1	2	3	
15	Anxious	0	1	2	3	
16	Repeatedly checks time	0	1	2	3	
17	Easily distracted	0	1	2	3	
18	Lacks energy	0	1	2	3	
19	Avoids others, spends much of time alone	0	1	2	3	
20	Easily upset if ritualistic behaviors are interrupted	0	1	2	3	
21	Lacks emotional facial expression	0	1	2	3	

22	Has shown difficulty in initiating routine tasks	0	1	2	3	
23	Listless	0	1	2	3	
24	Experiences panic attacks	0	1	2	3	
25	Avoids eye contact	0	1	2	3	
26	Trembles when frightening situations are not present	0	1	2	3	
27	Avoids peers	0	1	2	3	
28	Tearful	0	1	2	3	
29	Mood changes rapidly	0	1	2	3	
30	Sweats excessively	0	1	2	3	
31	More difficulty falling asleep than usual	0	1	2	3	
32	Clingy	0	1	2	3	
33	Wakes more easily than usual	0	1	2	3	
34	Agitated	0	1	2	3	
35	Avoids confined places	0	1	2	3	
36	Irritable	0	1	2	3	
37	Decreased appetite	0	1	2	3	
38	Increased communication	0	1	2	3	
39	Indecisive	0	1	2	3	
40	Decreased communication	0	1	2	3	
41	Increased laughter compared to usual	0	1	2	3	
42	Increased smiling compared to usual	0	1	2	3	
43	Fearful of specific items (specify)	0	1	2	3	
44	Outbursts of anger	0	1	2	3	

45	Exaggerated startle	0	1	2	3	
46	Excessive fidgeting compared to baseline	0	1	2	3	
47	Paces or wanders aimlessly	0	1	2	3	
48	Makes more eye contact than usual, i.e. is trying to get someone's attention	0	1	2	3	
49	Waits near a window	0	1	2	3	
50	Vomits	0	1	2	3	
51	Shows aggression to others (hitting, slapping, lashing out, grabbing), specify:	0	1	2	3	
52	Yelling or screaming	0	1	2	3	
53	Lies down on the ground and refuses to cooperate	0	1	2	3	
54	Injures self	0	1	2	3	
56	Engages in self-stimulating activities, such as head-banging	0	1	2	3	
57	Gags	0	1	2	3	
58	Disrobes (removes clothes)	0	1	2	3	
59	Refuses to eat	0	1	2	3	
60	Spits or drools to excess	0	1	2	3	
61	Pinches	0	1	2	3	
62	Pulls hair	0	1	2	3	

63	Bites or tries to bite someone else	0	1	2	3	
64	Eats excessively	0	1	2	3	
65	Bites nails	0	1	2	3	
66	Picks skin	0	1	2	3	
67	Cries excessively	0	1	2	3	
68	Other, please specify	0	1	2	3	
69	Other, please specify	0	1	2	3	
70	Other, please specify	0	1	2	3	

Does the person with AS have a preference for one caregiver (over others)? ___Yes ___No

If "yes," please explain: ____

____

____

Does the person with AS display agitation upon someone coming between them and their preferred caregiver? ___Yes ___No *If "yes," please explain:* ______________________

____

____

Does the person with AS display agitation if the preferred caregiver attends to someone else or attempts to leave for any amount of time? ___Yes ___No *If "yes," please explain:*

____

____

Does the preferred caregiver experience anxiety/fear when leaving the person with AS? ___Yes ___No *If "yes," please explain:* ____

Does the person with AS display agitation upon breaking gaze (eye contact) with the preferred caregiver? ___Yes ___No *If "yes," please explain:*
